# Invasive Corridor of Clivus Extension in Pituitary Adenoma: Bony Anatomic Consideration, Surgical Outcome and Technical Nuances

**DOI:** 10.3389/fonc.2021.689943

**Published:** 2021-06-25

**Authors:** Xiao Wu, Han Ding, Le Yang, Xuan Chu, Shenhao Xie, Youyuan Bao, Jie Wu, Youqing Yang, Lin Zhou, Minde Li, Shao Yang Li, Bin Tang, Limin Xiao, Chunlong Zhong, Liang Liang, Tao Hong

**Affiliations:** ^1^ Department of Neurosurgery, The First Affiliated Hospital of Nanchang University, Nanchang, China; ^2^ Department of Anatomy, School of Basic Medical Sciences, Anhui Medical University, Hefei, China; ^3^ Department of Neurosurgery, Shanghai East Hospital, Tongji University School of Medicine, Shanghai, China

**Keywords:** pituitary adenoma, clival invasion, endonasal endoscopic approach, epoxy sheet plastination, corridor, cancellous bone, anatomy

## Abstract

**Background:**

It is well known that the clivus is composed of abundant cancellous bone and is often invaded by pituitary adenoma (PA), but the range of these cancellous bone corridors is unknown. In addition, we found that PA with clivus invasion is sometimes accompanied by petrous apex invasion, so we speculated that the petrous apex tumor originated from the clivus cancellous bone corridor. The aim of this study was to test this hypothesis by investigating the bony anatomy associated with PA with clival invasion and its clinical significance.

**Methods:**

Twenty-two cadaveric heads were used in the anatomical study to research the bony architecture of the clivus and petrous apex, including six injected specimens for microsurgical dissection and sixteen cadavers for epoxy sheet plastination. The surgical videos and outcomes of PA with clival invasion in our single center were also retrospectively reviewed.

**Results:**

The hypoglossal canal and internal acoustic meatus are composed of bone canals surrounded by cortical bone. The cancellous corridor within clivus starts from the sellar or sphenoid sinus floor and extends downward, bypassing the hypoglossal canal and finally reaching the occipital condyle and the medial edge of the jugular foramen. Interestingly, we found that the cancellous bone of the clivus was connected with that of the petrous apex through petroclival fissure extending to the medial margin of the internal acoustic meatus instead of a separating cortical bone between them as it should be. It is satisfactory that the anatomical outcomes of the cancellous corridor and the path of PA with clival invasion observed intraoperatively are completely consistent. In the retrospective cohort of 49 PA patients, the clival component was completely resected in 44 (89.8%), and only five (10.2%) patients in the early-stage had partial residual cases in the inferior clivus.

**Conclusion:**

The petrous apex invasion of PA is caused by the tumor invading the clivus and crossing the petroclival fissure along the cancellous bone corridor. PA invade the clivus along the cancellous bone corridor and can also cross the hypoglossal canal to the occipital condyle. This clival invasion pattern presented here deepens our understanding of the invasive characteristics of PA.

## Introduction

Pituitary adenoma (PA) is a common benign tumor, but it has the biological characteristics of malignant tumors that invade adjacent structures, such as the sphenoid sinus (SS), cavernous sinus, suprasellar region, nasopharynx, and clivus (1). Cavernous sinus invasion is considered to be almost equivalent to the invasiveness of PA, until recently, still considered to be surgically challenging (2–5). Nevertheless, tumor invasiveness has many aspects, and clival invasion is also worthy of our attention.

The clivus used to be considered an unusual location for PA invasion, and most of the reported cases involving the clivus were ectopic PA cases (6–9). However, Chen et al. analyzed clival invasion with multidetector CT in 390 pituitary macroadenomas, and 32 (8.21%) patients had clival invasion (10). It is common knowledge that the clivus has a cancellous bone that is often invaded by PA. However, there are few studies on the clivus corridor of PA, including how the tumor extends into the clivus and the extent of cancellous bone involvement. In some cases, PA invading the clivus may be accompanied by petrous apex invasion, while petrous apex invasion is rarely seen in cases without clivus involvement. Therefore, we hypothesize that tumors in the petrous apex originate from the cancellous bone corridor within the clivus.

In this study, we studied the bony architecture of the clivus and petrous apex, including the extent and characteristics of the cancellous bone, by performing microsurgical dissection and a recently developed epoxy sheet plastination technique. We also reviewed 49 cases of PA involving the clivus treated by the endoscopic endonasal approach (EEA) in our institution. Finally, information useful for eventual clinical applications, including surgical techniques and outcomes, were discussed.

## Materials and Methods

### Microsurgical Dissection

The research was authorized by the Ethics Committee of Nanchang University. Six injected adult cadaveric specimens (12 sides) were used to dissect the bony architectures of the clivus and petrous apex (magnification ×4–40, surgical microscopes by Zeiss). The heads were fixed in a Mayfield head holder to maintain a stable position for navigation (Brainlab, Germany) and drilling (NSK, Japan). The specimens were bisected axially using a high-speed electric saw for stepwise superior-to-inferior and medial-to-lateral dissection in the clivus.

### Epoxy Sheet Plastination

Sixteen cadaveric heads with thirty-two sides underwent epoxy sheet plastination. The cadavers were donated for anatomic education and research to the Department of Anatomy at Anhui Medical University. The skull base tissue blocks were removed from the cadaver and plastinated by the E12/E6/E600 resin (Biodur, Heidelburg, Germany) ultrathin plastination technique (11). These undecalcified resin blocks were serially sectioned in the axial (eight sets), coronal (eight sets), and sagittal (16 sets) planes with the Exakt 310 CP cutting system (Exakt, Norderstedt, Germany). All sections stained with Stevenel’s blue and Alisarin red S were examined and photographed by a Leica DM6 B light microscope (Leica, Bensheim, Germany). This step was performed to better understand the relationship between the bony architectures of the clivus and the surrounding important neurovascular structures, including the internal carotid artery, petrous apex, hypoglossal nerve canal, and occipital condyle.

### Patient Population

The data of 49 PAs with clival invasion who underwent EEA between July 2015 and May 2020 in our single center were retrospectively reviewed, including 31 females and 18 males, with an average age of 49.1 years. The collection of medical records and surgical videos was authorized by the Institutional Review Board of Nanchang University. The patient’s age, sex, Knosp grade, pathological type, and extent of tumor resection were obtained from the medical records. Because the corridor of PA with upper clivus (dorsum sellae) invasion is not consistent with the middle and inferior clivus, it often directly invades the bony structures of the posterior clinoid process and dorsum sellae. In our study, we will not discuss this type of invasive PA.

### Preoperative Evaluation

All patients underwent conventional T1- and T2-weighted MRI scans and T1-weighted 3D MP-RAGE sequence scans before the operation. Moreover, CT scans were performed to obtain detailed information on the bony structures. In the preoperative radiological evaluation, we evaluated the upper, middle, and lower thirds of the clivus in the sagittal plane. The upper clivus extends from the posterior clinoid process to the sellar floor, the middle clivus extends from the sellar floor to the roof of the choana, and the lower clivus extends from the roof of the choana to the foramen magnum, corresponding to the nasopharyngeal clivus (12).

### Preoperative Preparation

In addition to routine examinations for PAs (visual acuity, visual field, endocrine examination), evaluations of the paraclival and petrous segments of the internal carotid artery (ICA) were performed. Under exceptional circumstances, evaluations of collateral circulation were also performed, and a balloon occlusion test was performed when necessary. Moreover, for PA with extensive clival invasion, we paid attention to cranial nerve-related symptoms such as abducens, acoustic and hypoglossal nerve-related symptoms.

### Assessment of the Path of PA Invading Clivus

The CT bone window was used to evaluate the type of SS pneumatization. The type of SS pneumatization was determined by the method proposed by Hammer et al. (13). A surgical video was used to further confirm the type of SS pneumatization and assess the relationship between SS pneumatization and clival invasion.

### Postoperative Imaging Evaluation

All patients were reexamined with MRI on the 3rd day after the operation. To minimize interrater variability, the extent of resection was assessed by two independent neurosurgeons in the First Affiliated Hospital of Nanchang University according to the postoperative enhanced MRI and CT thin-section data. We defined gross total resection as the absence of a residual tumor, subtotal resection as ≥80% of the tumor being resected, and partial resection as <80% of the tumor being resected.

## Results

### Microsurgical Dissection

Cancellous bone within clivus is composed of irregular grids, which are separated by bony septa and resemble honeycomb-like structures. The bony septa separating these grids are very thin and weak and are easily penetrated by the spatula into the next grid. These characteristics are obviously beneficial for the extension of the clival corridor in PA. However, we observed that the bony canal of hypoglossal nerve is composed of cortical bone. Therefore, the extent of cancellous bone within the clivus extend across the hypoglossal canal and reach the occipital condyle and the medial edge of the jugular foramen.

**Figure 1:**
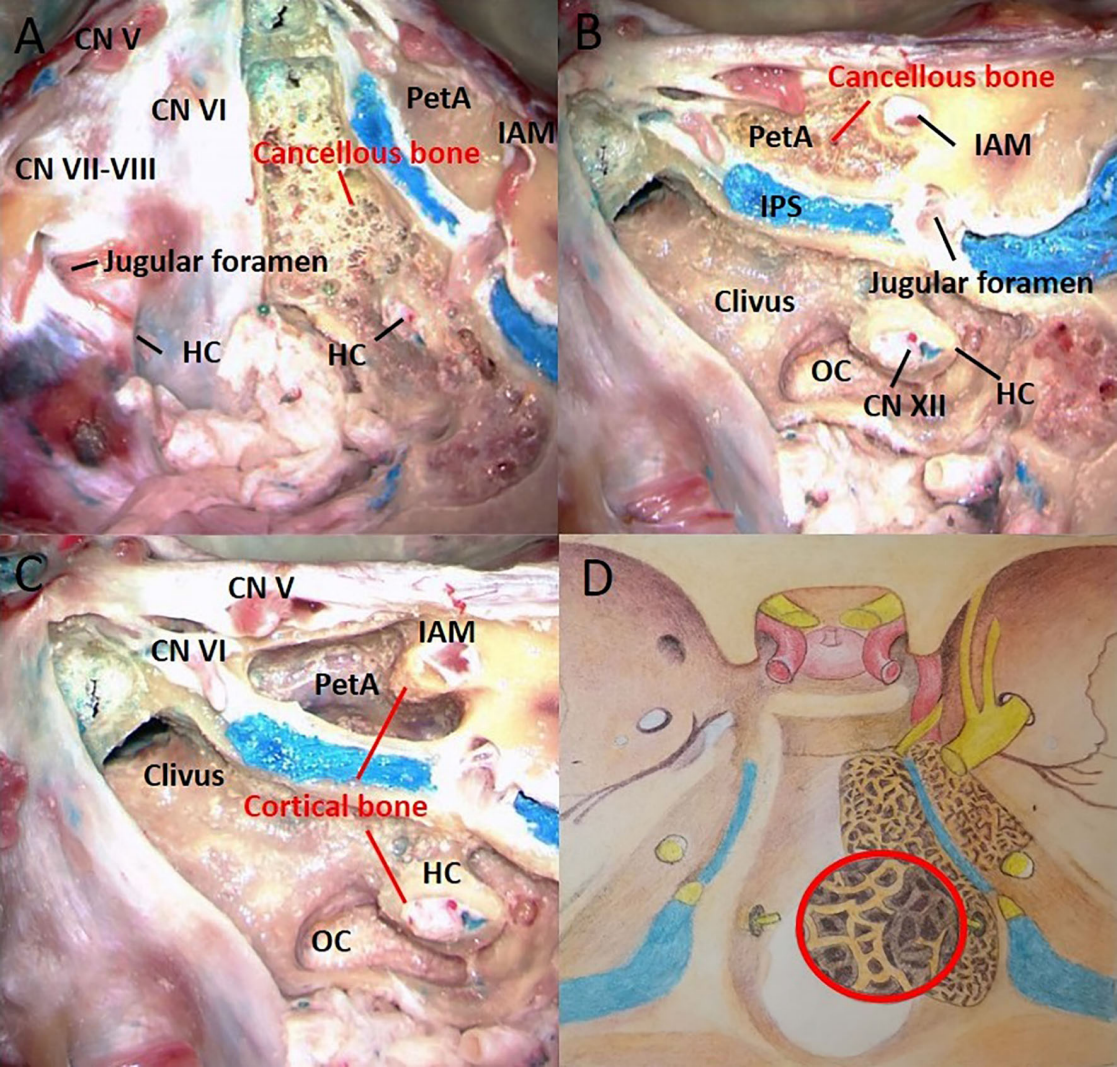
The right half of the cortical bone was removed, and the left half was used as the control. The bony architecture in the clivus and petrous apex includes the cancellous bone and cortical bone. **(A)** The clivus is made of loose cancellous bone beneath the cortical bone. **(B)** The cancellous bone within the clivus extends downward to the occipital condyle. The petrous apex is also made of loose cancellous bone beneath the cortical bone. **(C)** The bony canals of the acoustic and hypoglossal nerves are composed of cortical bone. **(D)** Artistic illustration demonstrating the shape and extent of cancellous bone in the clivus and petrous apex. The red circle represents the enlarged view of the cancellous bone structure. CN, cranial nerve; HC, hypoglossal canal; IPS, inferior petrous sinus; IAM, internal acoustic meatus; OC, occipital condyle; PetA, Petrous apex.

The bony architecture of the petrous apex is similar to that of the clivus. Interestingly, we found that the cancellous bones of the clivus and petrous apex were connected through petroclival fissure, and no tough cortical bone between them was found. Due to the bony canals of the acoustic nerve is composed of cortical bone, the cancellous bone corridor of the petrous apex only reached the medial edge of the internal acoustic meatus (**Figure 1**).

### Epoxy Sheet Plastination

Most of the microanatomy results are consistent with those of plastination anatomy studies. The bony surface of the clivus and petrous apex is composed of dense cortical bone, while the inner part is composed of loose cancellous bone separated by numerous thin bony septa. Dense cortical bone was not found between the petrous apex and the clivus, which indicated that PA can easily extend to the petrous apex through the clival cancellous corridor. Moreover, the bone canal of the petrous ICA surrounded by cancellous bone is relatively thin and weak, thereby careful manipulation is needed to protect the petrous ICA during tumor resection of the petrous apex component

**Figure 2:**
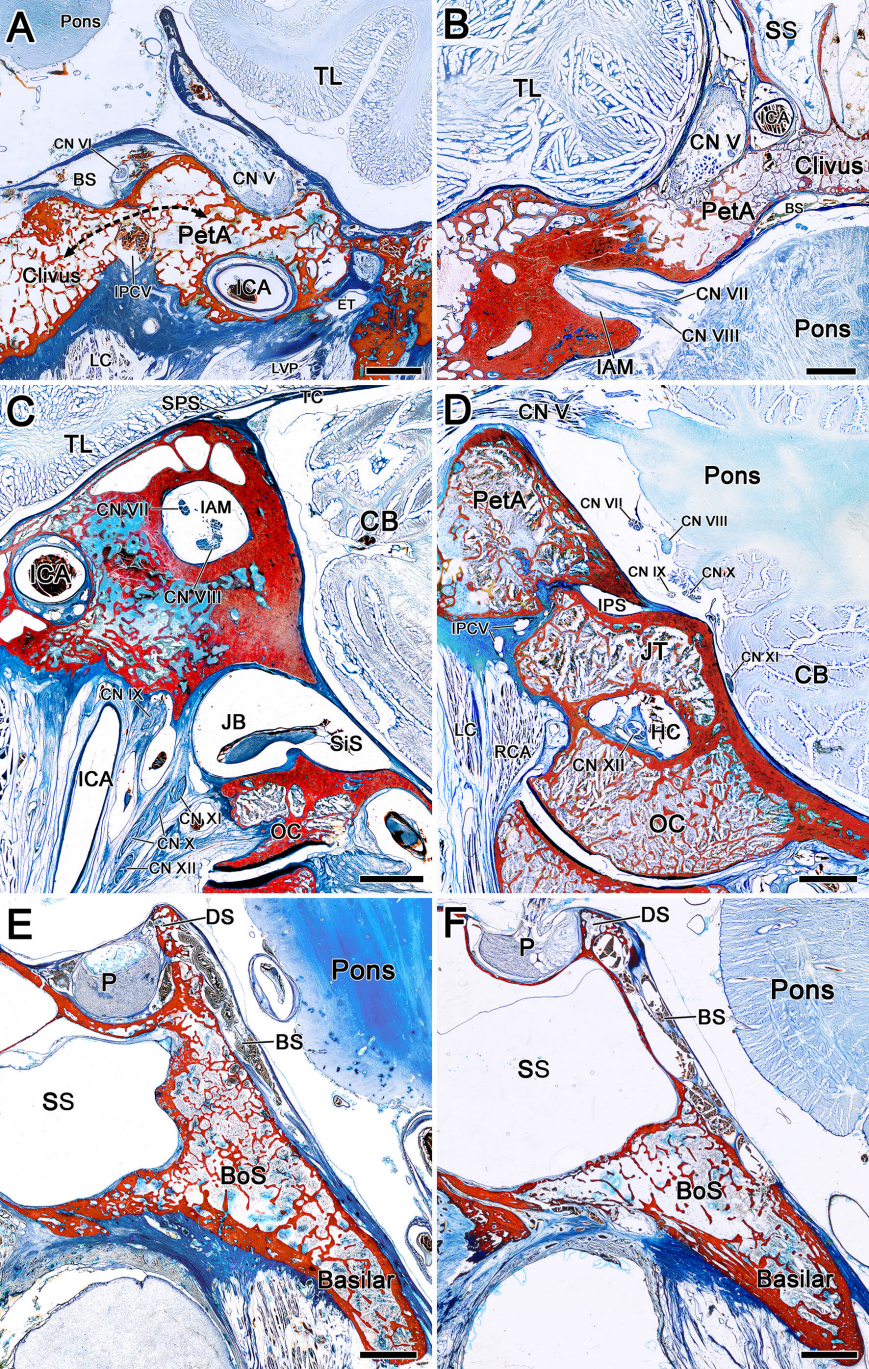
The composition and connection of bony architecture around the clivus were observed by epoxy sheet plastination. **(A)** Coronal sections of a right clivus and petrous apex. The bony surface of the clivus and petrous apex is composed of dense cortical bone, while the inner part is composed of loose cancellous bone separated by numerous thin bony septa. The bone canal of the petrous ICA surrounded by cancellous bone is relatively thin and weak. The black arrow indicates the communication of cancellous bone between the clivus and petrous apex. **(B)** Axial sections of a left clivus and petrous apex. The cancellous bone of the clivus communicates with that of the petrous apex to the medial margin of the IAM. **(C)** Sagittal sections of a right IAM. There is high density cortical bone around the IAM. **(D)** Sagittal sections of a right HC. The bony canal of the hypoglossal nerve is composed of cortical bone. **(E, F)** Sagittal section of clivus and sella turcica. There is no dense cortical bone between the body of sphenoid bone and the basilar part of occipital bone, and communicate through cancellous bone. The bony structure of upper clivus (dorsum sellae) can be mainly composed of cortical bone **(E)**, or the outer layer is surrounded by cortical bone, while the inner part is full of cancellous bone **(F)**. BS, basilar sinus; Basilar, basilar part of occipital bone; BoS, body of sphenoid bone; CB, cerebellum; DS, dorsum sellae; ET, eustachian tube; ICA, internal carotid artery; IPCV, inferior petroclival vein; IPS, inferior petrosal sinus; JB, jugular bulb; JT, jugular tubercle; LVP, levator veli palatini; LC, Longus capitis; MA, maxillary artery; NP, nasopharynx; PP, pterygoid plexus; P, pituitary; RCA, rectus capitis anterior; SPS, superior petrosal sinus; SiS, sigmoid sinus; SS, sphenoidal sinus; TC, tentorium cerebelli; TJ, temporomandibular joint. TL, temporal lobe. Bar = 5 mm.

Clivus is formed by the synostosis of the sphenoid bone and the basilar part of occipital bone. It was found that the bony structure of spheno-ocipital synchondrosis was similar to petroclival fissure, and there was no dense cortical bone to separate it, but communicated through cancellous bone, which also became the anatomical corridor for PA to enter the lower clivus. The bony structure of upper clivus is relatively complex, which can be mainly composed of cortical bone, or the outer layer is surrounded by cortical bone, while the inner part is full of cancellous bone (**Figure 2**).

### Relationship Between SS Pneumatization and Clival Invasion

**Figure 3:**
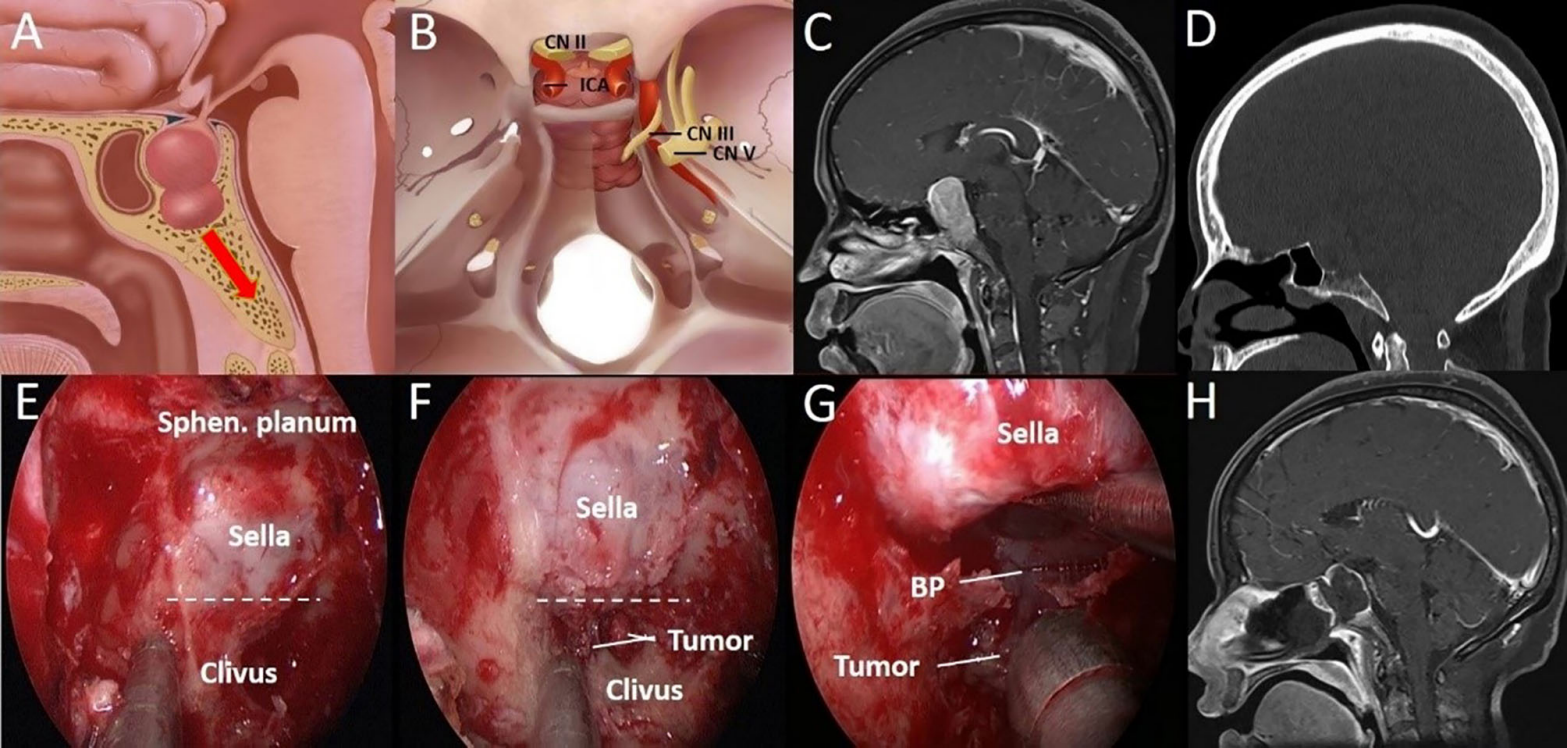
A 44-year-old woman with binocular vision blurred and headache for 2 years was aggravated for 10 days. **(A)** Artistic illustration demonstrating that the tumor directly invades the clivus through the sellar floor on sagittal view. **(B)** Intracranial view of PA with clival invasion. **(C)** Preoperative sagittal T1-weighted MRI shows PA with a daughter tumor in the clivus. **(D)** The CT bone window shows a presellar SS. The bony structure was destroyed to the middle clivus by tumor invasion. **(E)** After completion of the sphenoidotomy, SS is confirmed as presellar type under an endoscopic view. **(F)** The daughter tumor was exposed after drilling off the bony structure in the clivus recess. **(G)** Through the sellar floor, the breakthrough point of tumor invasion to the clivus can be seen directly under a close-up view. **(H)** Postoperative T1-weighted MRI shows complete resection of the tumor in the clivus. The dotted line indicates the location of the sellar floor. BP, Breakthrough point; CN, Cranial nerve; ICA, Internal carotid artery; Sphen, Sphenoid.

**Figure 4:**
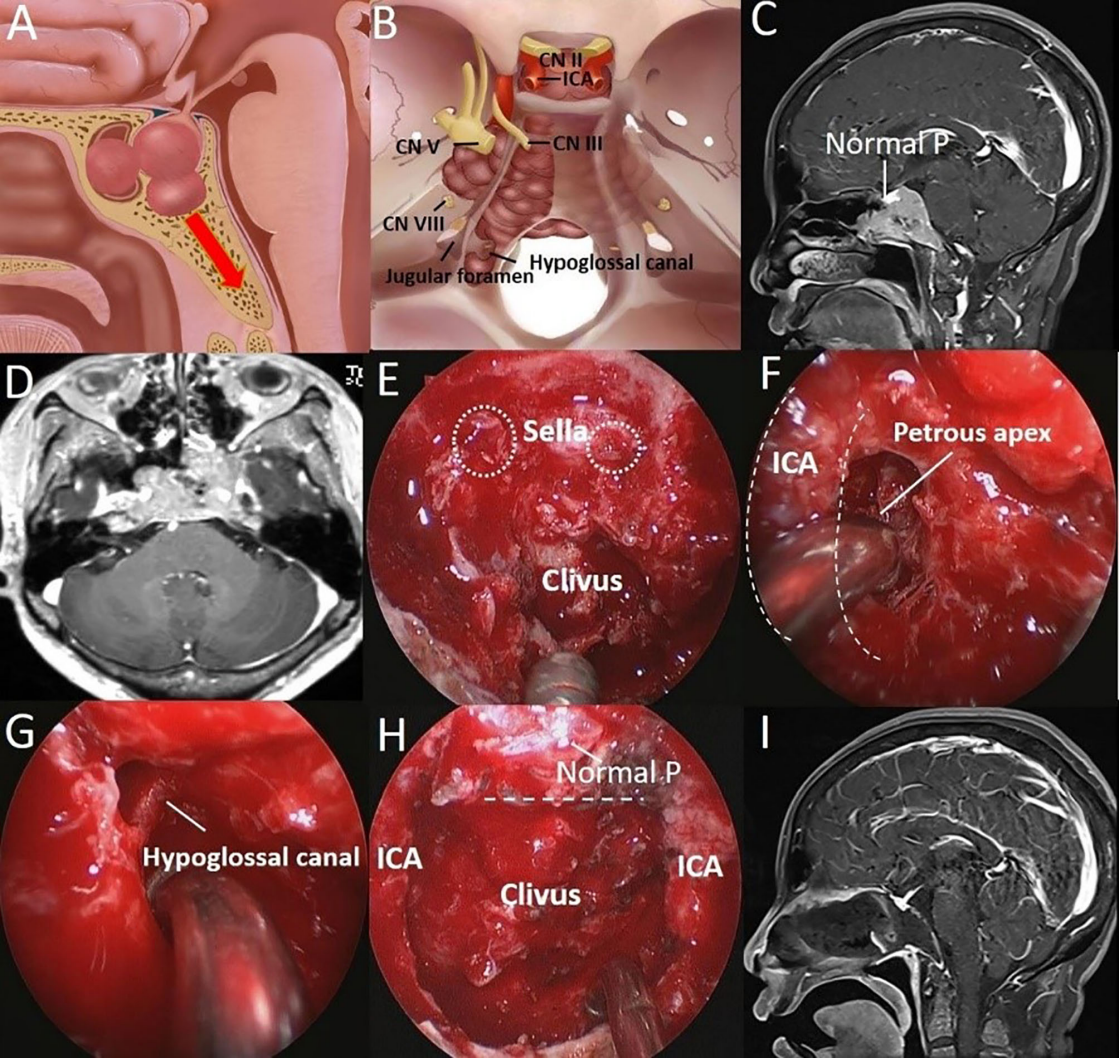
A 51-year-old woman was hospitalized 8 years after surgery for PA, and tumor recurrence was found for 1 year. **(A)** Artistic illustration demonstrating PA with clival and SS invasion simultaneously in presellar SS on sagittal view. **(B)** Intracranial view of PA extending laterally to the petrous apex and downward to the hypoglossal canal. **(C)** Preoperative sagittal T1-weighted MRI shows a PA extending to the inferior clivus, and the normal pituitary can be seen above the tumor. **(D)** Preoperative axial T1-weighted MRI shows a tumor with bilateral petrous apex extension. **(E)** After completion of the sphenoidotomy, SS is confirmed as presellar type under an endoscopic view. The breakthrough point of the PA from the anterior sellar wall into the SS can be seen. (white dotted circle). **(F)** The tumor (petrous apex component) was resected through the posterior space of the paraclival ICA (dotted curved line) under a 30° endoscope. **(G)** The tumor around the hypoglossal canal was resected under a 30° endoscope. **(H)** The tumor was completely resected, and the normal pituitary was well protected. The dotted line indicates the location of the sellar floor. **(I)** Postoperative T1-weighted MRI shows complete resection of the tumor in the clivus.

The CT bone window can be used to determine the type of SS and the corridors of the invading clivus. Conchal-type SS was not found in our case series due to its rarity. For patients with medium SS pneumatization, that is, the presellar type, PA can directly invade the clivus through the sellar floor (**Figure 3A**). In other cases, clival invasion may be accompanied by SS invasion, and PA can also invade the clivus through the SS floor (**Figure 4A**).

**Figure 5:**
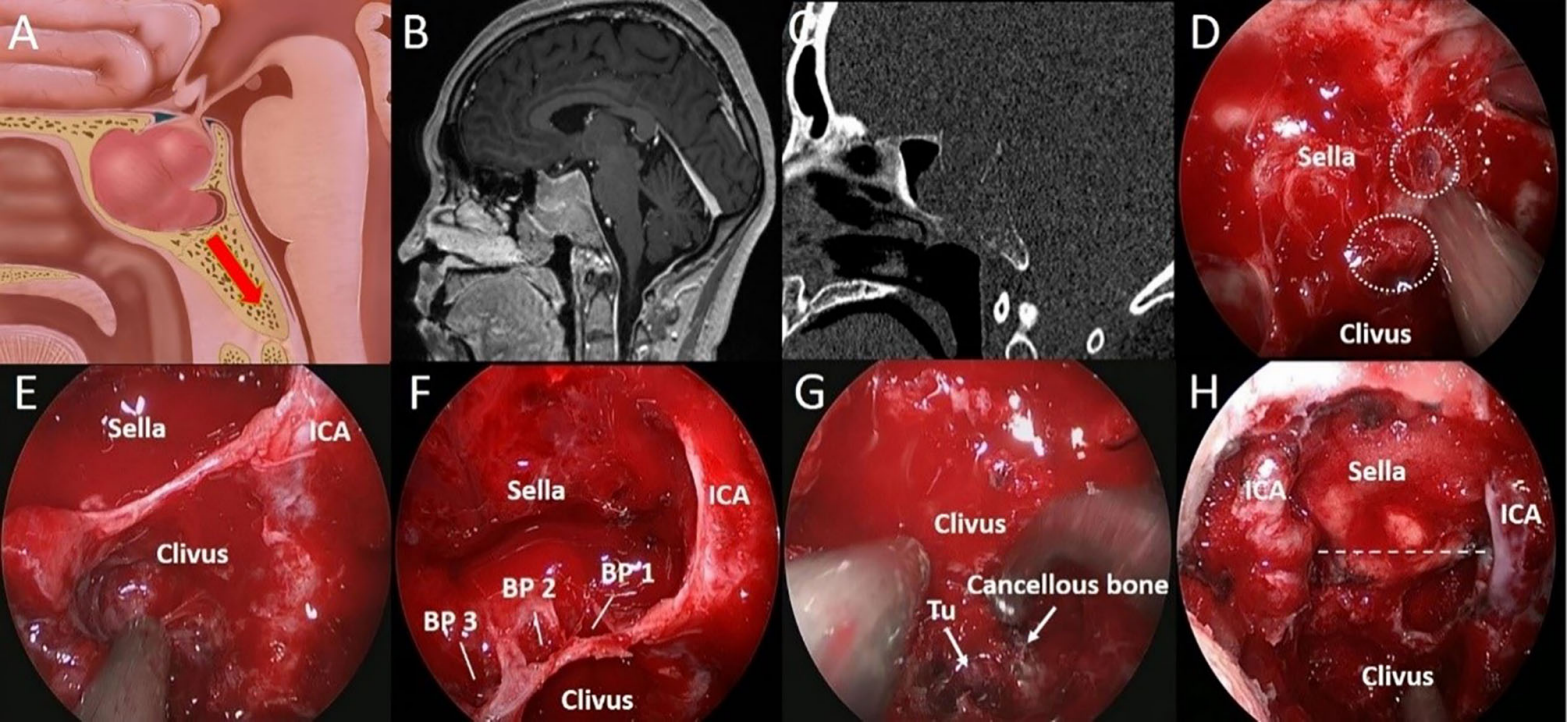
A 37-year-old woman with binocular vision blurred for 3 months. **(A)** Artistic illustration demonstrating that the PA invades the clivus through the SS floor with sufficient pneumatization on sagittal view. **(B)** Preoperative sagittal T1-weighted MRI shows PA with SS and clival invasion. **(C)** The bone window image of CT shows that SS pneumatization is sellar type. The bony structure was destroyed to the inferior clivus by tumor invasion. **(D)** After resecting the SS component, the breakthrough point of the PA from the anterior sellar wall into the SS can be seen. (white dotted circle). **(E)** Intraoperative observation confirmed that the SS was sellar type. In addition, the tumors in the middle clivus on MRI were all in the SS. **(F)** After tumor resection in the sellar region, it was found that there were three breakthrough points in the sellar floor, which led to downward invasion. **(G)** Intraoperative observation showed that the tumor (Tu) was hidden in the honeycomb-like structure formed by the cancellous bone within the clivus. **(H)** The tumor was completely resected, and the bilateral ICAs were exposed in the surgical field. The dotted line indicates the location of the sellar floor.

For patients with sufficient SS pneumatization, that is, the sellar type, the tumor can break through the sellar floor into the SS and then further invade the bone within the clivus through the SS corridor (**Figure 5A**).

### Surgical Techniques

All operations were performed under intraoperative electrophysiological monitoring. The surgical approach and preparation process are described in a previous report (14). Two surgeons (four hands) performed the surgery *via* the binostril EEA. A wide bilateral sphenoidotomy was performed to provide more access for better observation of the tumor invasion corridors. Intraoperatively, the tumor was found to be hidden and mixed in the honeycomb-like structure formed by the cancellous bone within the clivus. The extent of tumor invasion was consistent with the distribution of cancellous bone found in our microanatomy and epoxy sheet plastination study. Tumor resection cannot be performed with only the double suction technique. Tumors mixed with the cancellous bone should be removed together with a power drill or a curette. The cortical bone surrounding the clival surface often indicate the boundary of total tumor resection.

For patients with medium SS pneumatization, the cortical bone in front of the clivus was exposed as much as possible. Then, we looked for defects in the sellar floor after drilling the cortical bone in front of the clivus and removing the tumor along the natural corridor. This type of procedure is relatively safe because the tumor can be completely resected without exposing the ICA (**Figure 3**). If the patient also exhibits SS invasion, ICA protection should be taken seriously due to the risk of the erosion of bone covering the paraclival ICA (**Figure 4**).

However, in cases of tumors invading the clivus after breaking through the anterior sellar wall or sellar floor to enter the SS, paraclival ICA protection should be considered carefully during tumor resection. When tumors near the paraclival ICA (SS component) were resected, the ICA was often exposed in the SS. At this time, stripper should be used to carefully separate the boundary between ICA and PA to avoid blindly pulling tumor. In these cases, Doppler ultrasound or neuronavigation should be used to accurately locate the ICA (**Figure 5**).

Different landmarks may be used for tumors with varying extents of invasion in varying directions. The isolated hypoglossal canal was observed when a PA extended to the inferior clivus after the tumor was completely resected (**Figure 4G**). PAs can even cross the hypoglossal canal and reach the occipital condyle or the medial margin of the jugular foramen. Moreover, the medial edge of the internal acoustic meatus can be seen when PAs cross the petroclival fissure to reach the petrous apex, and special attention should be paid to protect the abducens nerve and petrous ICA during tumor resection. A sign of total tumor resection is total removal of the PA and cancellous bone within the clivus. Except for the bone canal of the hypoglossal nerve, an empty clivus was observed (**Figure 4H**). In fact, the endoscopic endonasal anatomy of these anatomical structures has been reported in the literature (15–17). It is necessary to be familiar with these anatomical characteristics to completely remove the tumor without neurovascular injury.

### Surgical Outcomes

The mean follow-up time was 29.5 months (range 5–58 months). Of the 49 tumors, 35 (71.4%) were nonfunctional adenomas, and 14 (28.6%) were functional adenomas. According to the Knosp classification system (18), 33 (67.3%) cases were classified as grade 4 cases, and 16 (32.7%) cases were classified as grade 1–3 cases.

Thirty-one (63.3%) patients had presellar SS with clival invasion, including eight (16.3%) patients without SS invasion and 23 (46.9%) patients with SS invasion. Ten (20.4%) patients had sellar SS. In addition, SS was difficult to identify in 8 patients, mainly due to the patient having a history of endoscopic surgery or extensive skull base destruction caused by giant PA invasion.

Regarding the extent of clival invasion, 22 (44.9%) patients had middle clival invasion, and 27 (55.1%) had inferior clival invasion. The intraoperative assessment and postoperative MRI findings on the extent of tumor resection showed that 31 (63.3%) patients underwent gross total resection, 15 (30.6%) patients underwent subtotal resection, and three (6.1%) patients underwent partial resection. In 44 (89.8%) PA patients, the clival component was completely resected, and only five (10.2%) patients in the early stage had partial residual cases in the inferior clivus (**Table 1**).

**Table 1:** Clinical characteristics of 49 PA with clival invasion.

Variables	No. of Patients
**Gender**	
Female	31 (63.3%)
Male	18 (36.7%)
**Age (years)**	49.1
**Pathological types**	
Nonfunctional	35 (71.4%)
Functional	14 (28.6%)
**Knosp grade**	
4	33 (67.3%)
1–3	16 (32.7%)
**Extent of clival invasion**	
Middle	22 (44.9%)
Inferior	27 (55.1%)
**Extent of resection (Entire PA)**	
GTR	31 (63.3%)
STR	15 (30.6%)
PR	3 (6.1%)
**Extent of resection (Clival component)**	44 (89.8%)
GTR	5 (10.2%)
STR	29.5 (5–58) M
**Mean follow-up time (Range)**	

GTR, gross-total resection; STR, sub-total resection; PR, partial resection; M, month.

Two patients had transient abducens nerve palsy, and no complications, such as auditory nerve or hypoglossal nerve injury, occurred. One (1/31) of the patients with total resection had recurrence at the last follow-up. Of the 18 patients who underwent subtotal or partial resection, nine underwent postoperative radiotherapy, and five underwent reoperation. At the last follow-up, there were five cases of recurrence.

## Discussion

According to the biological behavior of the tumor, PAs can be considered noninvasive PAs, invasive PAs or pituitary cancer. The concept of invasive PA was first proposed by Jefferson in 1940 and refers to the condition in which PA cells invade the surrounding structures and destroy corresponding tissues (19). Among the types of invasive PA, clival invasion is easily ignored, but it is worthy of further study.

### Characteristics of PA With Clival Invasion

The clivus is an endochondral bone; its progression involves cartilaginous formation followed by reabsorption preceding bone deposition (20, 21). At birth, the clivus is composed of partially ossified basioccipital and basisphenoid parts separated by the spheno-occipital synchondrosis. PA with clival involvement has been reported for many years (10, 22, 23); however, the growth corridors of clival invasion and corresponding surgical techniques have not been delineated. With exploration of clival invasion corridors, a high total resection rate has been achieved in our center.

The characteristics of clival invasion in PA are obvious and unique. Because PAs often extends along the loose cancellous corridor without cortical bone erosion, tumor growth in the clivus remains within the bone cavity and is restricted by tough cortical bone. Therefore, the configuration of the clivus bone in radiological images does not appear abnormal, whereas other tumors such as chordoma and osteogenic tumors are often accompanied by cortical bone invasion and distort the appearance of the clivus bone.

The clival component is different from any other invasive region. Intraoperatively, we found that PA in the sellar, SS, suprasellar region and cavernous sinus were easily aspirated as a whole by a suction device. In contrast, the tumors in the clivus were separated and mixed by numerous tiny and paper-like bone septa and hidden in the honeycomb-like cancellous bone. Therefore, it was difficult to achieve complete resection because it was difficult to aspirate the tumors by a suction device alone. Residual tumors can cause recurrence and a wider extent of invasion, leading to the need for more complex treatment.

### Clivus–Petrous Apex Corridor for PA Invasion

In this study, we researched the relationship between the cancellous bone corridor and PA invasion using microsurgical dissection and the recently developed epoxy sheet plastination technique. The plastination technique uses durable and transparent resin to replace water and fat in tissues and cells, thus keeping all the neural and vascular structures in their natural state *in situ* without decalcification (11, 24, 25). The combination of plastination and microsurgical dissection is a good way to explore the extent of cancellous bone within the clivus. In contrast to other anatomical studies on the clivus, we studied the bony architecture, which helped us better understand the characteristics of PA invasion.

Structurally, cancellous bone is composed of numerous grids of different shapes and sizes, separated by paper-like bony septa and partially communicated, which promotes PA extension. The cancellous bone within cortical bone extends downward from the sellar or SS floor to the occipital condyle and to the medial edge of the jugular foramen. Laterally, the cancellous bone of the clivus and petrous apex are connected through petroclival fissure extending to the medial edge of the internal acoustic meatus. Fortunately, the extent of cancellous bone found in the anatomical study mentioned above was almost the same as that of clival tumor invasion observed during the operation. Obviously, the petrous apex invasion of PA is caused by the tumor invading the clivus and crossing the petroclival fissure along the cancellous bone corridor. Therefore, the results support our hypothesis that the PA with petrous apex invasion is originate from the corridor of cancellous bone within the clivus. This finding is of great significance for selecting the appropriate surgical techniques because it is difficult to achieve total resection with craniotomy or the EEA if the petrous apex corridor is considered to be a separate corridor.

However, because the medial edge of the internal acoustic meatus is composed of tough and dense cortical bone, the petrous apex corridor does not cross it and extend laterally. For downward clival invasion, although the tumor can reach the occipital condyle, it will not erode the bone canal of the hypoglossal nerve. Our anatomic results showed that cortical bone formed around those two bone canals, which explains our intraoperative findings.

### SS Pneumatization Affects Clival Invasion

PA extending into the bony structure within the clivus is related to the pneumatization of SS. The SS is located in the body of sphenoid bone, and its pneumatization forms the main sinus cavity occupying the sphenoid body. In cases of well pneumatization, it can extend to the dorsum sellae and clivus, and even to greater and lesser wings, anterior clinoid process and pterygoid process (26).

According to the degree of pneumatization, there are various classification for the types of SS. Hammer et al. classified in the sagittal plane as three types: conchal, presellar, and sellar (13). Based on the correlation of endoscopic skull base surgery, Vaezi et al. classified SS pneumatization into three types: previdian, intercanal, and postrotundum (27). Wang et al. classified SS into the six basic types based on the direction of pneumatization: sphenoid body, lateral, clival, lesser wing, anterior, and combined (26). The most widely adopted classification is proposed by Hammer, which is also used in this study.

Different types of SS require different surgical techniques and intraoperative precautions. For conchal-type SS, we believe that the corridor of clival invasion directly extends into the clivus without SS invasion. Among the cases in our study, pressellar SS with both clivus and SS invasion was the most common type (46.9%, 23/49). This kind of tumor invades the clivus extensively, reaching all longitudinal regions of the cancellous bone within the clivus.

In patients with sellar SS, PAs can extend to the SS first and then erode the clivus through the SS floor. However, this phenomenon is relatively rare because the tumor always grows along the direction of the least resistance, and tumors more commonly fill the SS and then protrude into the nasal cavity. Most importantly, according to our experience, this type of tumor often invades the bony structure, which covers the paraclival ICA, so the operation should be performed carefully.

**Figure 6:**
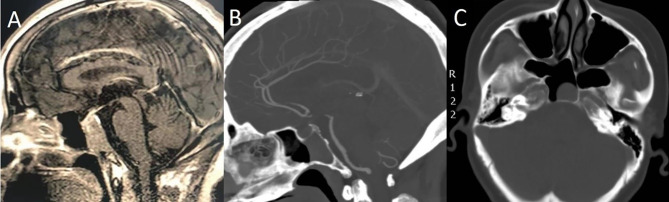
A 55-year-old woman with headache for 4 months. **(A)** Preoperative sagittal T1-weighted MRI shows PA with clival invasion. **(B)** Sagittal CT bone window shows that the patient had sufficient SS pneumatization. The cortical bone of the SS floor was intact, and the tumors were all in the SS without clival bony erosion. **(C)** Axial CT bone window shows that the tumor was completely located in the SS.

Furthermore, cases of sufficient SS pneumatization that extends to the inferior clivus can be mistaken as cases of clivus invasion preoperatively, but in fact, these tumors are located in the SS without clival bony erosion. Therefore, we should carefully determine whether the cortical bone of the SS floor is invaded by a PA with preoperative CT bone window imaging (**Figure 6**).

### Surgical Approaches

In traditional craniotomy, the upper clivus can be approached through the orbitozygomatic route or the variant approach; the middle clivus can be reached through the transpetrosal approach; and the lower clivus can be accessed through the far-lateral approach. Due to the proximity of the clivus to critical neurovascular structures, clival surgery is associated with a high rate of postoperative complications and recurrences (28).

In most cases, PA with clival invasion is located in only the epidural space and does not require a transcranial procedure. With the advanced EEA, the resection of clival lesions is relatively easy since this approach allows direct visualization and exposes the ventral side of the whole clivus without intracranial manipulation (29–31). The natural corridor of tumor invasion from the sellar to the clivus can be fully exposed with the EEA, which plays a significant role in total tumor resection. Therefore, the EEA should be considered the preferred approach for this type of tumor.

### Limitations

Most of the cases in our study had multidirectional invasion, and few tumors invaded only the clivus, which is more representative. Preoperative CT bone window imaging can be used to predict the locations of corridors, but this process is difficult in patients with a history of endoscopic surgery and giant PA with extensive bone destruction of the skull base.

## Conclusion

We confirmed the intraoperative finding that PAs within the clivus was extend through the cancellous bone corridor by conducting microanatomic and plastination studies. PAs with petrous apex invasion are caused by the tumor invading the clivus and further crossing the petroclival fissure along the cancellous bone corridor. Moreover, we have also found that the degree of SS pneumatization has a large influence on clival invasion. These growth characteristics of clival invasion are different from those of tumors in other invasive regions, requiring different surgical techniques. On this basis, we will greatly improve the total resection rate of PA invading the clivus and reduce recurrence.

## Data Availability Statement

The original contributions presented in the study are included in the article/supplementary material. Further inquiries can be directed to the corresponding authors.

## Ethics Statement

The studies involving human participants were reviewed and approved by the Institutional Review Board of Nanchang University. The patients/participants provided their written informed consent to participate in this study.

## Author Contributions

TH and LL: study concept and design. XW, HD, LY and XC: acquisition of data. SX, YB, and JW: analysis and interpretation of data. YY, LZ, ML, SL, BT, LX and CZ: critical revision of manuscript. All authors contributed to the article and approved the submitted version.

## Funding

This work was supported by the National Natural Science Foundation of China (grant nos. 82060246 and 31500967), Natural Science Foundation of the Anhui Higher Education Institutions of China (No. KJ2020A0145).

## Conflict of Interest

The authors declare that the research was conducted in the absence of any commercial or financial relationships that could be construed as a potential conflict of interest.
